# THE IMPACT OF 12 WEEKS OF ADAPTED DANCE ON BALANCE, GAIT, AND LOWER EXTREMITY FUNCTION AMONG PERSONS WITH DEMENTIA

**DOI:** 10.1093/geroni/igac059.2788

**Published:** 2022-12-20

**Authors:** Keara Quijano, Crystal Bennett

**Affiliations:** University of West Florida, Milton, Florida, United States; University of West Florida, Lillian, Alabama, United States

## Abstract

Person’s living with dementia commonly experience difficulty with mobility. Difficulties with these activities can lead to an increased fall risk, resulting in an increased loss of independence. Dance engages various parts of the brain including the cerebellum that is primarily involved in coordinating balance, posture, body positioning, and voluntary movement. An aim of this study was to assess whether 12 weeks of adapted dance improves balance, usual walking speed, and lower extremity function among persons living with dementia. An experimental design was used to randomly assign persons with dementia to either a 12-week adapted dance or social stimulation group. The convenience sample consisted of 12 participants, ages ranging from 62–97 years. The adapted dance is low impact where one foot is always in contact with the floor and is appropriate for older adults with cognitive and physical limitations. At baseline and at 12 weeks, measures of balance, gait, and lower extremity function were assessed. From baseline to posttest, the dance group had greater increased times for maintaining tandem balance (+47.5%) and faster times for usual gait speeds (+15.1%); compared with the social stimulation group tandem balance time (+0.98%) and usual gait speed times (+10.5%). A limitation of this study is the small sample size.

